# Cervical Pain in Non-Chondrodystrophic Dogs: Associations with Clinical Onset, Neurological Group and Disease Category

**DOI:** 10.3390/ani16111673

**Published:** 2026-05-30

**Authors:** Domenico Fugazzotto, Marco Tabbì, Chiara Caterino, Girolamo Messina, Gaetano Principato, Simona Di Pietro, Claudia Giannetto, Gerardo Fatone, Francesco Macrì, Simone Minato

**Affiliations:** 1 Department of Veterinary Sciences, University of Messina, 98168 Messina, Italy; domenicofugazzotto@gmail.com (D.F.); messinagirolamo24@gmail.com (G.M.); gaetanoprincipato@gmail.com (G.P.); simona.dipietro@unime.it (S.D.P.); claudia.giannetto1@unime.it (C.G.); francesco.macri@unime.it (F.M.); 2 Ospedale Veterinario San Francesco, 31038 Castagnole, Italy; minatosimone81@gmail.com; 3 Department of Veterinary Medicine and Animal Production, University of Naples “Federico II”, 80137 Naples, Italy; chiara.caterino@unina.it (C.C.); fatone@unina.it (G.F.)

**Keywords:** dog, non-chondrodystrophic breed, cervical pain, clinical onset, magnetic resonance imaging

## Abstract

The information available on cervical myelopathies (CMs) and cervical pain (CP) in non-chondrodystrophic breeds remains limited compared to chondrodystrophic breeds. The expression of CP in CMs may depend on lesion type, severity and onset of clinical signs. This retrospective single-centre study reviewed the medical records of 112 purebred non-chondrodystrophic dogs referred for neurological evaluation, in which C1-T2 lesions were subsequently confirmed by magnetic resonance imaging (MRI). Clinical onset was categorised as acute (≤7 days) or chronic (>7 days). Neurological grade was classified into four neurological groups (G1–G4). CP was graded using a study-specific, non-validated five-point ordinal clinical scale. Multifactorial analysis of variance (ANOVA), odds ratios with 95% confidence intervals, and multivariable binomial logistic regression were performed. ANOVA showed significant effects of group (*p* < 0.0001), disease category (*p* < 0.05), and clinical onset (*p* < 0.02) on CP scores. Post-hoc comparison showed significantly lower CP scores in chronic onset than in acute onset (*p* < 0.0001). Acute onset was associated with CP in G2 (OR = 71.0; *p* < 0.0002) and remained associated with CP after adjustment for groups (adjusted OR = 4.42; 95% CI: 1.28–15.28; *p* = 0.019). These findings suggest that clinical onset may contribute to CP expression in non-chondrodystrophic dogs with CMs. Acute onset, particularly in ambulatory myelopathic patients, appears more frequently associated with CP. However, due to the retrospective design, the heterogeneous diagnostic composition, and the study-specific nature of the CP score, these associations should be interpreted cautiously.

## Simple Summary

Cervical myelopathies (CMs) are common clinical and neurological findings in dogs that can result from a variety of conditions. The relationship between cervical pain (CP) expression and lesion type, neurological severity and clinical onset, in non-chondrodystrophic dogs with CMs is not well understood. This retrospective study examined 112 purebred non-chondrodystrophic dogs with C1-T2 lesions diagnosed by magnetic resonance imaging to investigate whether acute onset was more often associated with CP than chronic onset. The study found that acute onsets were more frequently associated with CP, especially in ambulatory dogs. However, some non-ambulatory dogs showed less CP despite having severe neurological dysfunction. Moreover, different disease categories were distributed differently across neurological groups. The results of this study suggest that clinical onset may influence pain expression, whereas the severity of neurological signs and disease categories may influence the clinical manifestation of CP. Overall, recognition of this association may support clinical prioritization, diagnostic imaging and therapeutic decision-making in dogs affected by CMs.

## 1. Introduction

Cervical pain (CP) and cervical myelopathies (CMs) are common clinical and neurological findings in dogs that can result from a variety of conditions, including trauma, intervertebral disc disease (IVDD), inflammatory and infectious disorders, neoplasia, and structural, developmental, or degenerative anomalies [1,2,3,4]. Among these, IVDDs are considered one of the most frequent CMs, with an estimated lifetime prevalence in dogs of 2–3.5% [5,6]. Although many IVDDs have been extensively characterised in both chondrodystrophic and non-chondrodystrophic breeds, current knowledge regarding other CMs in non-chondrodystrophic dogs remains comparatively limited [6,7,8,9,10]. Cervical neurological signs attributable to CMs are commonly observed in non-chondrodystrophic breeds and, similarly to chondrodystrophic breeds, may result from a wide range of disorders, including hydrated nucleus pulposus extrusion (HNPE) [11,12,13,14], acute non-compressive nucleus pulposus extrusion (ANNPE) [15,16,17], fibrocartilaginous embolism (FCE) [18,19], meningoencephalitis of unknown etiology (MUE) [20,21], steroid-responsive meningitis-arteritis (SRMA) [22,23], cervical spondylomyelopathy (CSM) [24,25,26], syringomyelia [27], and neoplastic disease [28,29,30]. However, the relative prevalence of these conditions and their associated clinical features remains poorly characterised in non-chondrodystrophic breeds.

Mechanisms leading to CP can be broadly classified as mechanical, neuropathic, or secondary to disorders outside the cervical region [31]. The clinical expression of CP in dogs with CMs is multifactorial, depending on lesion type, neurological severity, and duration of clinical signs [1]. Acute disc extrusion and inflammatory disorders are commonly associated with CP, likely due to sudden mechanical compression, meningeal irritation, and local release of inflammatory mediators [22,23,32]. In contrast, certain acute non-compressive disc events, such as HNPE or ANNPE, may lead to marked neurological deficits with minimal or absent CP, possibly reflecting differences in epidural inflammation and mechanical stress [11,13,14,15,16,17,18,19]. Similarly, chronic compressive or intramedullary disorders may induce adaptive or maladaptive neuroplastic changes within spinal nociceptive pathways, potentially modifying pain perception over time [33,34,35,36,37]. Several retrospective studies have reported the prevalence of CP in specific breeds or disease categories, but few have specifically evaluated the relationship between onset of clinical signs and pain expression in non-chondrodystrophic dogs with CMs [1,38,39]. To the authors’ knowledge, onset-dependent CP expression has not been systematically evaluated across neurological groups and disease categories in this population. It remains unclear whether an acute onset of neurological signs is more consistently associated with CP than with chronic disease, particularly across different neurological groups. Separate evaluation of non-chondrodystrophic dogs is warranted because differences in body size, cervical biomechanics, IVDD phenotype, and disease spectrum may influence both neurological presentation and CP expression. This population appears to show a broader and more heterogeneous spectrum of CMs than chondrodystrophic breeds, making direct extrapolation from the latter or from breed-specific cohorts inappropriate [7,8,9,10]. A clearer understanding of pain expression in non-chondrodystrophic dogs with CMs may improve diagnostic and therapeutic decision-making.

This single-centre retrospective study aimed to characterise the underlying causes and clinical presentation of CMs in non-chondrodystrophic dogs and to assess the association between the temporal onset of neurological signs and CP expression. It was hypothesized that CP expression would differ according to clinical onset, neurological group, and disease category.

## 2. Materials and Methods

### 2.1. Study Design and Case Selection

The medical records of client-owned dogs referred for neurological evaluation to San Francesco Veterinary Hospital between January 2022 and December 2025 were retrospectively reviewed. Only purebred non-chondrodystrophic dogs with neurological findings consistent with a C1-T2 spinal cord lesion and that had undergone cervical magnetic resonance imaging (MRI) were included. Signalment (breed, age, sex, and body weight) was recorded for each patient. A definitive or presumptive diagnosis was required based primarily on MRI findings, with or without ancillary diagnostic testing. Chondrodystrophic breeds and mixed-breed dogs, as well as cases with missing onset history, missing CP assessment, or uncertain or multifocal neuroanatomical localisation were excluded. Cases that had received analgesic, anti-inflammatory, or corticosteroid treatment before referral were also excluded to avoid potential interference with CP assessment. Written informed consent for diagnostic procedures and use of anonymised clinical data for research purposes was obtained from all owners at admission.

### 2.2. Neurological Assessment, Cervical Pain Evaluation, and Clinical Onset

Neurological examinations and CP assessment were performed by a European College of Veterinary Neurology (ECVN) Resident before sedation, analgesia, or administration of any MRI-related medication. Neurological status was classified into four clinical groups using a modified grading scheme derived from the neurological severity scale described by Sharp and Wheeler [40]. Dogs in Group 1 (G1) exhibited CP in the absence of neurological deficits. Group 2 (G2) included dogs with ambulatory tetraparesis, hemiparesis, or thoracic limb monoparesis. Group 3 (G3) included dogs with non-ambulatory tetraparesis or tetraplegia with preserved deep pain perception. Group 4 (G4) included dogs with tetraplegia, absent deep pain perception, and/or evidence of respiratory compromise (as in loss of spontaneous ventilation).

The presence of CP was determined based on neurological examination findings, including pain elicited during cervical palpation or manipulation. CP was considered present when at least one of the following signs was documented during neurological examination: low head carriage; reduced cervical range of motion and/or resistance during flexion, extension, or lateral flexion of the neck; vocalisation during cervical manipulation or palpation of the cervical epaxial musculature; twitching of the cervical musculature; or caudal flinching of the ears during cervical palpation or manipulation, according to previously published criteria [1,39]. CP severity was additionally graded using a study-specific 5-point ordinal clinical scale ranging from 0 to 4, based on the overall severity of the clinical signs observed during neurological examination. The final score was assigned according to the most severe reproducible sign observed during examination. Grade 0 indicated absence of cervical pain, with normal head carriage, normal cervical range of motion, and no resistance or pain response during cervical palpation or manipulation. Grade 1 indicated minimal cervical discomfort, defined as slightly low head carriage and/or mild cervical stiffness, with preserved cervical range of motion and only minimal resistance to manipulation, without overt pain responses such as vocalization, muscle twitching, or ear flinching. Grade 2 indicated mild-to-moderate cervical pain, defined as low head carriage with mildly to moderately reduced cervical range of motion and consistent but tolerable resistance to manipulation, without overt vocal pain responses. Grade 3 indicated moderate-to-severe cervical pain, defined as low head carriage with clearly reduced cervical range of motion and marked resistance to manipulation, together with reproducible non-vocal pain responses such as cervical muscle twitching, guarding, or caudal ear flinching during palpation or manipulation. Grade 4 indicated severe cervical pain, defined as an overt pain response during cervical palpation or manipulation, including vocalization and/or inability to complete gentle cervical examination because of marked pain or guarding. Reduced cervical range of motion and low head carriage were typically present. To improve reproducibility, resistance to cervical manipulation was interpreted as follows: minimal resistance indicated that the examination could be completed without interruption; consistent resistance indicated that manipulation was tolerated but clearly opposed; and marked resistance indicated that manipulation was substantially limited or interrupted by guarding or pain response. Only reproducible findings were considered for score assignment. The duration of clinical signs was based on clinical history obtained at presentation. Clinical onset of signs was classified as acute if the duration of clinical signs was 7 days or less, and as chronic if signs had been present for more than 7 days. A 7-day cutoff was chosen to differentiate acute clinical presentations from chronic ones, based on pragmatic clinical criteria commonly applied in neurological practice [41].

### 2.3. MRI and Diagnostic Classification

All MRI examinations were performed using a 0.4 Tesla open magnet system (Hitachi Aperto Lucent, Tokyo, Japan). The imaging protocol was based on previously published guidelines proposed by the Canine Spinal Cord Injury Consortium (CANSORT-SCI) [42]. Each patient was positioned in dorsal or sternal recumbency with non-magnetic foam positioning aids to maintain straight spinal alignment. Images were acquired in sagittal, transverse, and dorsal planes using T2-weighted (T2W), T1-weighted (T1W), and short tau inversion recovery (STIR) sequences. Post-contrast T1W sequences were obtained after intravenous administration of gadodiamide (Omniscan^®^, GE Healthcare S.r.l., Milan, Italy; 0.1 mmol/kg) when clinically indicated. Slice thickness ranged from 2 to 3 mm, depending on the size of the patient. MRI studies were interpreted by an ECVN Diplomate, who was blinded to the neurological examination findings and CP assessment results, using a DICOM viewer (Horos Project™, Version 3.0, Purview, Annapolis, MD, USA). Diagnoses were established based on MRI findings and ancillary diagnostic testing, when available. Cases were categorised as intervertebral disc herniation (IVDH), particularly intervertebral disc extrusion (IVDE) and intervertebral disc protrusion (IVDP), HNPE, ANNPE, CSM, MUE, SRMA, neoplastic disease, infectious disease, or other less common etiologies, according to previously described criteria [3,8,11,15,16,17,21,22,33]. Cerebrospinal fluid (CSF) analysis was performed in all cases with suspected inflammatory or infectious disease, based on clinical and MRI findings. Abnormal results were defined according to established reference values for nucleated cell count and total protein concentration [43]. However, since no histopathological confirmation was obtained, all inflammatory diagnoses were classified as presumptive rather than definitive.

### 2.4. Statistical Analysis

Descriptive statistics were calculated for all variables. Categorical data were expressed as absolute numbers and percentages. For exploratory analyses, the main diagnoses were also regrouped into broader biologically coherent disease categories: degenerative disease including IVDE, HNPE, ANNPE, and disc-associated (DA-CSM), inflammatory disease including MUE and SRMA, and neoplastic disease. Infectious and other etiologies were not included in exploratory inferential subgroup analyses because of their low numbers and heterogeneity. CP score was considered the primary outcome of the exploratory analysis, and multifactorial analysis of variance (ANOVA) was performed to evaluate the effects of group, disease category, and clinical onset. The CP score was treated as a semi-quantitative clinical variable for the purpose of this exploratory score-based analysis. Data were expressed as mean ± standard error (SE). Bonferroni’s test was applied for post-hoc comparisons. In addition, CP was also analysed as a dichotomous dependent variable (present/absent), while clinical onset category (acute vs. chronic) was treated as the primary explanatory variable. The association between clinical onset category and the presence of CP was assessed using contingency table analysis, applying either the chi-square test or Fisher’s exact test as appropriate according to expected cell counts. Odds ratios (ORs) with 95% confidence intervals (CIs) were calculated to estimate the strength of the association between acute clinical onset and CP in G2 and G3. Dogs in G1 were excluded to avoid introducing definitional bias since CP was part of the group definition, and dogs in G4 were excluded due to the small sample size, precluding meaningful statistical comparison. Separate analyses were performed for G2, G3, and G2-G3 combined, as predefined subgroup comparisons. An additional comparison evaluated differences in CP prevalence between G2 and G3. To further assess whether clinical onset was associated with the binary presence of CP after adjustment for group, a multivariable binomial logistic regression model was fitted in G2 and G3. CP status (present/absent) was entered as the dependent variable, while clinical onset and group were entered as categorical explanatory variables. Chronic onset and G2 were used as reference categories. Adjusted odds ratios (ORs) with 95% CIs were calculated. Disease category was not included in the multivariable logistic regression model because of sparse data and quasi-complete separation in some diagnostic categories. An additional sensitivity analysis was performed in the same G2-G3 population after exclusion of SRMA cases to assess whether the association between clinical onset and CP remained consistent after removal of this potentially influential painful inflammatory subgroup. A multivariable binomial logistic regression model was fitted in this reduced population using the same dependent and explanatory variables and the same reference categories as in the primary model. Statistical significance was set at *p* < 0.05. All analyses were performed using Stata version 14.0 (StataCorp, College Station, TX, USA).

## 3. Results

### 3.1. Study Population

A total of 112 purebred non-chondrodystrophic dogs met the inclusion criteria.

**Figure 1:**
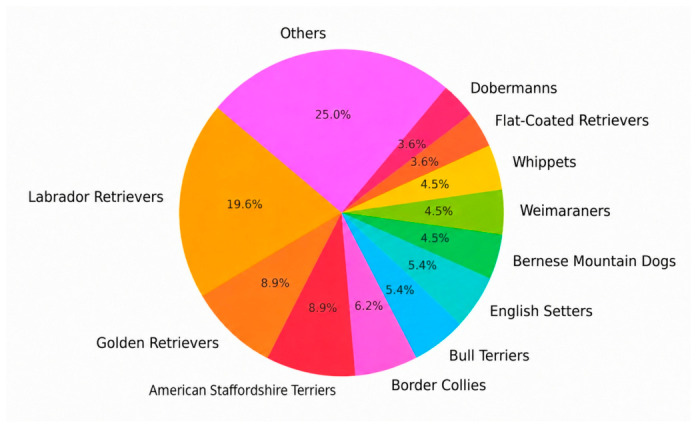
Breed distribution of non-chondrodystrophic dogs included in the study. The breeds included in the “Others” category are detailed in Supplementary Table S1.

The mean age of the study population was 82.6 ± 39.2 months. Of these, 58 (51.8%) were male and 54 (48.2%) were female. The most common breeds were Labrador Retriever (22/112; 19.6%), Golden Retriever (10/112; 8.9%), and American Staffordshire Terrier (10/112; 8.9%), while several other medium- and large-breed dogs were represented in smaller numbers (Figure 1).

Based on the clinical history obtained at presentation, 89 dogs (79.5%) were classified as having acute onset of clinical signs (≤7 days), whereas 23 dogs (20.5%) were classified as having chronic onset (>7 days).

Thirty-seven (*n* = 37; 33.0%) dogs were included in G1. Forty-five (*n* = 45; 40.2%) dogs were included in G2, and twenty-eight (*n* = 28, 25.0%) in G3. Two (*n* = 2; 1.8%) dogs were included in G4.

### 3.2. Diagnostic Distribution Across Neurological Groups

The most frequent diagnosis was IVDH (44/112; 39.3%), followed by inflammatory diseases (35/112; 31.3%), neoplastic conditions (16/112; 14.3%), DA-CSM (7/112; 6.3%), infectious diseases (7/112; 6.3%), and other etiologies (3/112; 2.7%). Among IVDHs, the most affected intervertebral space was C3-C4 (18/44; 40.9%), followed by C4-C5 (12/44; 27.4%), C5-C6 (6/44; 13.6%), C2-C3 (5/44; 11.3%) and C6-C7 (3/44; 6.8%). Among inflammatory diseases, eighteen (*n* = 18) were SRMA and seventeen (*n* = 17) were MUE. Among neoplastic lesions, extramedullary lesions were more prevalent (11/16; 68.8%) than intramedullary lesions (5/16; 31.3%).

**Figure 2:**
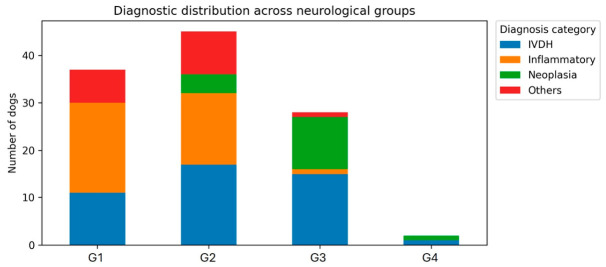
Disease distribution across groups (G1-G4). “Others” includes disc-associated cervical spondylomyelopathy (DA-CSM), infectious diseases, and other less common etiologies.

In G1, inflammatory diseases predominated (19/37; 51.4%), largely represented by SRMA (15/37; 40.5%) and less commonly MUE (4/37; 10.8%). IVDH accounted for 11/37 (29.7%) and all cases were consistent with cervical IVDE, most frequently involving C4-C5 (4/11; 36.4%), followed by C2-C3, C3-C4 and C5-C6 (each 2/11; 18.2%), and C6-C7 (1/11; 9.1%). Infectious diseases (6/37; 16.2%), including discospondylitis (4/37; 10.8%) and epidural empyema (2/37; 5.4%), and DA-CSM (1/37; 2.7%) were also present. In G2, IVDH remained the most frequent diagnosis (17/45; 37.8%), closely followed by inflammatory disease (15/45; 33.3%). DA-CSM (6/45; 13.3%), neoplastic conditions (4/45; 8.9%), infectious disease (1/45; 2.2%; epidural empyema), and other etiologies (2/45; 4.4%), including vertebral artery ectasia and a subarachnoid diverticulum, were also present. In G3 (*n* = 28), IVDH represented the most frequent diagnosis (15/28; 53.6%), whereas inflammatory disease was uncommon (1/28; 3.6%; MUE). Neoplastic conditions (11/28; 39.3%), including vertebral and intramedullary tumours, and other etiologies (1/28; 3.6%; FCE) were also present. Within the IVDH subgroup, cases were mainly consistent with HNPE (6/15; 40.0%), followed by IVDE (5/15; 33.3%) and ANNPE (4/15; 26.7%). In G4 one dog had IVDH (C4-C5 IVDE; 1/2; 50.0%) and the other had a neoplastic disease (1/2; 50.0%). No inflammatory or infectious cases were recorded in this group. Disease distribution across groups is summarised in Figure 2.

### 3.3. Cervical Pain Distribution and Association Analyses

In dogs classified as G1, CP was present in all cases (37/37; 100%). Among G2 dogs, CP was present in 40/45 cases (88.9%) and absent in 5/45 (11.1%). In G3 dogs, CP was present in 15/28 cases (53.6%) and absent in 13/28 (46.4%). The distribution of disease categories across groups is summarised in Table 1.

**Figure 3:**
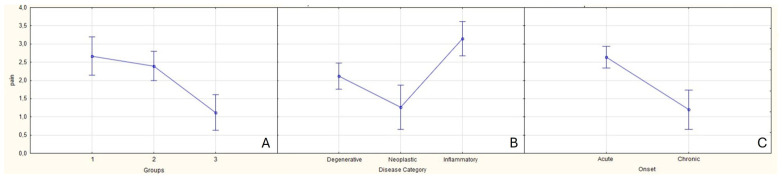
Least-squares mean plots derived from multifactorial ANOVA illustrating the effects of neurological group (**A**), disease category (**B**), and clinical onset (**C**) on CP scores.

After regrouping diagnoses into broader disease categories and excluding G4 dogs, infectious diseases, and other etiologies, one hundred (*n* = 100) dogs were included in the exploratory ANOVA-based analysis. The regrouped disease categories included degenerative disease (*n* = 50), inflammatory disease (*n* = 35), and neoplastic disease (*n* = 15). The application of multifactorial ANOVA showed a significant effect of group (G1-G3) (*p* < 0.0001), disease category (degenerative, inflammatory, and neoplastic disease) (*p* < 0.05), and clinical onset (acute, ≤7 days; chronic, >7 days) (*p* < 0.02) on CP scores. Bonferroni’s post-hoc comparison test showed significantly lower CP scores in G3 (1.11 ± 0.20) than in G1 (3.12 ± 0.20; *p* < 0.0001) and G2 (2.57 ± 0.20; *p* < 0.0001) (Figure 3A). Also, statistically significant differences in CP scores were observed among disease categories (Figure 3B). In particular, neoplastic disease had significantly lower CP scores (1.26 ± 0.28) than degenerative disease (2.12 ± 0.17) (*p* < 0.001) and inflammatory disease (3.14 ± 0.23) (*p* < 0.0001), and degenerative disease had significantly lower CP scores than inflammatory disease (*p* < 0.0001). Statistically significant differences in CP scores were also observed among clinical onset categories (Figure 3C), with significantly lower values in chronic onset (1.20 ± 0.25) than acute onset (2.63 ± 0.15) (*p* < 0.0001).

**Figure 4:**
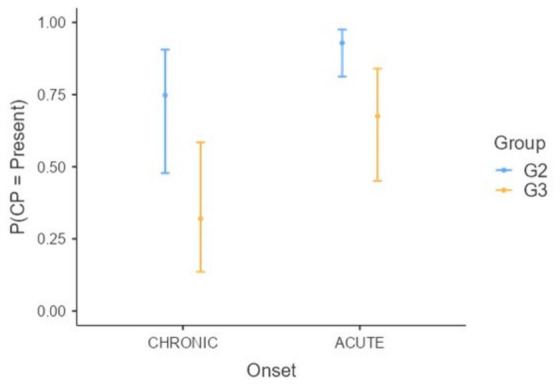
Multivariable binary logistic regression. Estimated probabilities of CP presence in G2 and G3 according to clinical onset and group. Vertical bars indicate 95% confidence intervals.

Among G2 dogs, acute clinical onset was significantly associated with the presence of CP (OR = 71.0; 95% CI: 3.43–1471.31; *p* < 0.0002). CP was observed in all dogs with acute onset, whereas it was present in 5 of 10 dogs with chronic onset. In G3 dogs, no significant association was identified between clinical onset category and CP (OR = 1.0; 95% CI: 0.20–4.29; *p* = 1.00). In this group, CP was present in 9 of 17 dogs with acute onset and 6 of 11 dogs with chronic onset. When G2 and G3 were analysed together, acute onset remained significantly associated with CP (OR = 5.0; 95% CI: 1.60–15.64; *p* < 0.006). Finally, comparison between groups demonstrated that G2 dogs were significantly more likely to exhibit CP than G3 dogs (OR = 6.93; 95% CI: 2.11–22.80; *p* < 0.001). In the multivariable binomial logistic regression model, acute onset was significantly associated with the presence of CP (adjusted OR = 4.42; 95% CI: 1.28–15.28; *p* = 0.019), whereas G3 was associated with lower odds of CP than G2 (adjusted OR = 0.16; 95% CI: 0.046–0.551; *p* = 0.004) (Figure 4).

In additional sensitivity analysis excluding SRMA cases from the same G2-G3 population, acute onset remained significantly associated with the presence of CP after adjustment for neurological group. In this multivariable binomial logistic regression model (*n* = 70), acute onset was associated with higher odds of CP compared with chronic onset (adjusted OR = 4.25; 95% CI: 1.23–14.66; *p* = 0.022), whereas G3 dogs showed lower odds of CP than G2 dogs (adjusted OR = 0.17; 95% CI: 0.05–0.58; *p* = 0.005).

## 4. Discussion

This retrospective cohort study examined 112 purebred non-chondrodystrophic dogs with CMs. The onset of clinical signs was associated with the presence of CP, particularly in the G2 subgroup, whereas this association was not evident within the G3 subgroup alone. Consistently, the exploratory CP score analysis showed an effect of both group and clinical onset on CP expression. These findings suggest that CP expression in CMs may vary according to neurological severity and clinical onset.

In our study, the most representative CMs with acute onset were IVDD and inflammatory diseases, particularly SRMA. Across groups, inflammatory disorders, particularly SRMA, prevailed in G1, IVDH prevailed in G2 and G3, and neoplastic disease became especially relevant in G3. This distribution may suggest that neurologically mild but more painful presentations (IVDE, SRMA) were more often associated with acute meningeal inflammation. Conversely, more severe non-ambulatory but less painful presentations (HNPE/ANNPE, DA-CSM, neoplasia) were more often associated with progressive neurological dysfunction. This pattern is broadly consistent with previous studies on the painful phenotype of SRMA, the clinicopathological features of HNPE, the presentation of spinal neoplasia, and the chronic compressive nature of DA-CSM [13,22,23,25,29,32,33,34,44,45,46,47,48,49,50,51].

The most common diagnosis in our study group was IVDH, accounting for 39.3% of cases. This finding is consistent with epidemiological data indicating that IVDD is a significant cause of cervical spinal cord dysfunction in dogs [1,5]. While non-chondrodystrophic breeds are generally considered to be less prone to acute disc extrusion than chondrodystrophic breeds [6,52], degenerative and disc-related disorders still account for a significant proportion of CMs in this group. In the present cohort, Golden Retriever was frequently represented among IVDE cases, whereas DA-CSM was mainly observed in Weimaraner and Dobermann. The predominance of the C3-C4 intervertebral space observed in our study is consistent with imaging-based investigations in selected breeds, including reports describing a similar tendency in certain brachycephalic breeds [7,10,53]. These anatomical patterns likely reflect the biomechanical loading and mobility characteristics specific to each spinal segment [54,55].

The association between clinical onset and CP expression is biologically plausible and is supported by previous literature on cervical spine disorders [55]. Acute compressive events, such as IVDE, cause the displacement of nucleus pulposus material into the vertebral canal. This sudden mechanical compression of the spinal cord and nerve roots is often accompanied by local inflammatory responses, vascular changes, and activation of nociceptive fibers within the meninges and epidural tissues [32,33,34,56]. The resulting meningeal irritation and radicular inflammation are major contributors to CP in acute disc disease [56,57]. A similar mechanism may be invoked for immune-mediated inflammatory conditions. In SRMA, for example, acute neutrophilic inflammation of the meninges produces marked CP, which is often the most prominent clinical sign [22,23]. As inflammation subsides with immunosuppressive treatment, pain usually improves, which highlights the close link between active meningeal inflammation and pain perception [58]. In the exploratory analysis, both disease category and clinical onset were associated with CP score, with higher scores observed in acute-onset than in chronic-onset cases. The observed association is therefore compatible with the pathophysiology of both mechanical and inflammatory acute CMs. Although these results may suggest the clinical relevance of temporal pattern, they should be interpreted as associative rather than as definitive evidence of an independent association between clinical onset and CP expression. This association may also partly reflect the predominance of disc-related (IVDE) and inflammatory diseases (SRMA) among acute presentations, rather than clinical onset alone. The very large OR observed in G2, together with its extremely wide CI, suggests instability related to sparse data or near-complete separation. Because disease category could not be included in the multivariable logistic regression model due to sparse cells and quasi-complete separation, residual confounding by heterogeneous disease composition cannot be excluded. Accordingly, clinical onset should be interpreted as a clinically useful temporal feature rather than as an independent determinant of CP expression. However, the adjusted logistic model and the sensitivity analysis excluding SRMA cases both supported the persistence of an association between acute onset and CP, suggesting that the association was not solely driven by the painful phenotype of this subgroup.

Likewise, the lower prevalence of CP in chronic cases may partly reflect differences in lesion type and temporal adaptive changes in spinal nociceptive pathways. In dogs, a spinal cord injury can damage the ascending nociceptive pathways, thus modifying the transmission and perception of pain signals and potentially influencing the way in which behavioural pain is expressed [35]. In the case of acute IVDH, the sudden mechanical rupture of innervated perispinal tissues, including the dura mater and the dorsal longitudinal ligament, causes pain [59,60]. This is followed by local inflammation, with the release of pro-algesic mediators that activate and sensitize nociceptors, producing peripheral sensitization and, if prolonged, central sensitization within the spinal cord [61]. As the injury stabilizes, inflammation enters a resolution phase, inflammatory mediator levels decrease, and both peripheral and central sensitization may regress [62]. As a result, pain may decrease significantly or disappear despite the persistence of structural injury, because the nociceptive cascade initiated by the acute event is no longer actively maintained, as peripheral sensitization may resolve once healing progresses [59]. In contrast, chronic IVDH is commonly characterized by annular protrusion, with disc material displacing progressively rather than abruptly, as occurs in nucleus pulposus extrusion. In these cases, chronic spinal cord compression, progressive demyelination or long-standing intramedullary disease can lead to structural and functional reorganization in the dorsal horn and supraspinal centers [35,36,37,59]. Experimental and clinical studies in veterinary and comparative medicine have described neuroplastic processes, including an altered excitatory-inhibitory balance and central sensitisation phenomena, that can modulate pain perception over time [35,59]. Furthermore, severe spinal cord injury can alter sensory processing by disrupting ascending tracts, thereby modifying nociceptive expression. Discrepancies between neurological severity and perceived pain have been reported in spinal cord injury and neuropathic pain models [35,36,59], suggesting that pain intensity does not necessarily correspond to the extent of motor impairment. In our study, G3 dogs were defined as non-ambulatory with tetraparesis or tetraplegia and preserved deep pain perception and therefore represent patients with substantial spinal cord dysfunction [32]. In these dogs, motor impairment and deficits in postural reactions may dominate the clinical picture, potentially masking or complicating the recognition of pain-related behaviours [13]. These mechanisms may explain both the lower prevalence of CP observed in chronic cases within our cohort and the lack of a significant association between clinical onset and CP in G3. The relatively small size of this subgroup, particularly among chronic G3 dogs, may have limited statistical power to detect a true association. A biological explanation is also plausible but remains speculative in the context of the present study. The way in which pain is expressed may also be influenced by the specific subtype of disc-related pathology. Compared with classic IVDH, HNPE and ANNPE have been reported to produce variable degrees of CP [11,12,13,14,15,16,17,18]. In HNPE, the extruded material typically consists of hydrated, minimally degenerated nucleus pulposus, which may transiently compress the nerve roots with limited epidural inflammation. Consequently, dogs may exhibit pronounced neurological deficits yet experience relatively mild or absent CP [13,14,33]. Within the G3 subgroup, disc-associated cases were mainly consistent with HNPE and ANNPE, conditions that may produce marked neurological dysfunction with limited or absent CP [11,12,13,14,15,16,17,18,19]. Therefore, the lack of a significant association in G3 may partly reflect the relatively high proportion of non-compressive IVDD with a less overt pain phenotype. These pathophysiological distinctions emphasise that pain expression depends not only on compression, but also on the inflammatory environment and the surrounding tissue response to the lesion and further support a cautious reading of pain expression in non-ambulatory dogs.

From a clinical standpoint, the findings of this study highlight the importance of carefully integrating the patient’s medical history into the neurological evaluation. In ambulatory dogs, whether with other cervical neurological signs or not, the presence of CP could suggest active compressive or inflammatory pathology. However, the absence of CP, particularly in chronic or more severely affected patients, should not lead to an underestimation of disease severity. Chronic CMs may present minimal overt CP despite significant structural compromise, emphasising the importance of thorough neurological examinations and appropriate diagnostic imaging, regardless of perceived discomfort [63].

The retrospective design of the study may have introduced potential biases, including reliance on owner-reported history for temporal classification. Referral bias may also have influenced the distribution of causes, resulting in an overrepresentation of more severe or complex cases, as well as disorders more likely to require specialist referral for advanced imaging or surgical treatment. Consequently, the case mix described here may not fully reflect the broader population of non-chondrodystrophic dogs with CMs encountered in primary-care or non-referral settings, thereby limiting the external validity and generalizability of our findings. Despite the inclusion of 112 cases overall, the small size of individual subgroups limited their representativeness and may have reduced the strength of subgroup-specific conclusions. As previously reported in studies on comparable disease categories [41,44,45,46,47,48,49,50,51], the 7-day cutoff used to distinguish acute from chronic onset was intended as a pragmatic clinical categorization rather than a strict biological boundary. Although this dichotomization cannot fully capture the gradual transition between acute and chronic disease, it enabled a retrospective classification to explore temporal differences in CP expression. The scale used in this study for CP assessment was a semi-quantitative clinical tool rather than a formally validated pain scoring system. Therefore, CP scores should be interpreted as clinical estimates rather than as objective measures of pain severity. In addition, the observed association between acute onset and CP may partially reflect the heterogeneous diagnostic composition of the study population rather than an independent temporal effect. Because different disease categories may differ substantially in both CP expression and temporal evolution, some degree of diagnosis-related confounding cannot be excluded. Although MRI review was blinded to the neurological examination findings and CP assessment results, both the clinical evaluation and MRI interpretation were performed by single observers, and therefore the reproducibility of these assessments could not be evaluated. Future multicenter prospective studies including larger study populations and advanced diagnostic imaging would help to clarify the relationship between clinical onset, neurological severity, and CP expression in canine CMs.

## 5. Conclusions

In non-chondrodystrophic dogs with CMs, acute onset was more frequently associated with CP, particularly in ambulatory patients, whereas chronic onset may be associated with limited CP despite severe structural pathology. The results of this study suggest that clinical onset may influence CP expression, whereas the severity of neurological signs and disease categories may influence the clinical manifestation of CP. Therefore, recognition of this association may support clinical prioritization, timely imaging, and therapeutic decision-making in dogs with CMs. These findings should, however, be interpreted in light of the retrospective design, the study-specific non-validated CP scoring system, and the heterogeneous diagnostic composition of the study population.

## Figures and Tables

**Table 1:** Distribution of disease categories across groups, further stratified by clinical onset and CP. Values are reported as number of dogs. AO: acute onset; CO: chronic onset; CP: cervical pain; NO-CP: absence of cervical pain. The distribution of CP scores according to specific diagnoses is provided in Supplementary Table S2.

Disease Category	G1		G2				G3				G4	
	AO	CO	AO		CO		AO		CO		AO	CO
	CP	CP	CP	NO-CP	CP	NO-CP	CP	NO-CP	CP	NO-CP	CP	NO-CP
**Degenerative**	12	0	17	0	3	3	7	7	1	0	1	0
**Inflammatory**	19	0	15	0	0	0	1	0	0	0	0	0
**Neoplastic**	0	0	1	0	2	1	1	0	5	5	0	1
**Infectious**	5	1	1	0	0	0	0	0	0	0	0	0
**Others**	0	0	1	0	0	1	0	1	0	0	0	0
**Total**	**36**	**1**	**35**	**0**	**5**	**5**	**9**	**8**	**6**	**5**	**1**	**1**

## Data Availability

The original contributions presented in this study are included in the article/Supplementary Materials. Further inquiries can be directed to the corresponding author.

## Supplementary Materials

The following supporting information can be downloaded at: https://www.mdpi.com/article/10.3390/ani16111673/s1, Table S1: Breeds included in the “Others” category of Figure 1; Table S2: Distribution of cervical pain (CP) scores according to specific diagnoses in the study population.

## Abbreviations

The following abbreviations are used in this manuscript:

CP	Cervical pain
CMs	Cervical myelopathies
IVDD	Intervertebral disc disease
HNPE	Hydrated nucleus pulposus extrusion
ANNPE	Acute non-compressive nucleus pulposus extrusion
FCE	Fibrocartilaginous embolism
MUE	Meningoencephalitis of unknown etiology
SRMA	Steroid-responsive meningitis-arteritis
CSM	Cervical spondylomyelopathy
MRI	Magnetic resonance imaging
ECVN	European College of Veterinary Neurology
IVDH	Intervertebral disc herniation
IVDE	Intervertebral disc extrusion
IVDP	Intervertebral disc protrusion
