# Supplementary material for: Cervical Pain in Non-Chondrodystrophic Dogs: Associations with Clinical Onset, Neurological Group and Disease Category
Source: Animals (Basel). 2026 May 30;16(11):1673. doi: 10.3390/ani16111673 (PMC13255795; doi:10.3390/ani16111673)
Supplement: Supplementary file 1 [file animals-16-01673-s001.zip › animals-4344471-supplementary.pdf]

Breed	n	%
Greyhound	3	2.7
Australian Shepherd	3	2.7
Boxer	3	2.7
Épagneul Breton	3	2.7
German Shepherd	3	2.7
Akita Inu	2	1.8
Rottweiler	2	1.8
Italian Pointer	2	1.8
Irish Setter	2	1.8
Shiba Inu	1	0.9
Dalmatian	1	0.9
Belgian Shepherd	1	0.9
Dogue de Bordeaux	1	0.9
Cane Corso	1	0.9
**Total**	**28**	**25.0**

Supplementary Table S1. Breeds included in the “Others” category of Figure 1. Values are reported as absolute numbers and percentages of the overall study population.

Diagnosis	n	CP score				
		**0**	**1**	**2**	**3**	**4**
IVDE	34	0	0	11	18	5
HNPE	6	3	1	2	0	0
ANNPE	4	4	0	0	0	0
DA-CSM	7	3	0	4	0	0
SRMA	18	0	0	0	0	18
MUE	17	0	10	0	0	7
Neoplastic disease	16	7	0	8	1	0
Discospondylitis	4	0	0	3	1	0
Epidural empyema	3	0	0	3	0	0
Vertebral artery ectasia	1	0	1	0	0	0
Subarachnoid diverticulum	1	1	0	0	0	0
FCE	1	1	0	0	0	0
**Total**	**112**	**19**	**12**	**31**	**20**	**30**

Supplementary Table S2. Distribution of cervical pain (CP) scores according to specific diagnoses in the study population. Values are reported as number of dogs.
